# Analysis of the Accelerator-Driven System Fuel Assembly during the Steam Generator Tube Rupture Accident

**DOI:** 10.3390/ma14081818

**Published:** 2021-04-07

**Authors:** Di-Si Wang, Bo Liu, Sheng Yang, Bin Xi, Long Gu, Jin-Yang Li, Janne Wallenius, You-Peng Zhang

**Affiliations:** 1 Institute of Modern Physics, Fudan University, Shanghai 200433, China; 20210200015@fudan.edu.cn (D.-S.W.); 20210200011@fudan.edu.cn (B.L.); 18210200009@fudan.edu.cn (S.Y.); 19110200027@fudan.edu.cn (B.X.); 2 Institute of Modern Physics, Chinese Academy of Sciences, Lanzhou 730000, China; lijinyang@impcas.ac.cn; 3 University of Chinese Academy of Sciences, Beijing 100049, China; 4 School of Nuclear Science and Technology, Lanzhou University, Lanzhou 730000, China; 5 KTH, Division of Nuclear Engineering, Albanova University Centre, 10691 Stockholm, Sweden; janne@neutron.kth.se

**Keywords:** accelerator-driven system, heavy liquid metal, CFD simulation, two-phase flow

## Abstract

China is developing an ADS (Accelerator-Driven System) research device named the China initiative accelerator-driven system (CiADS). When performing a safety analysis of this new proposed design, the core behavior during the steam generator tube rupture (SGTR) accident has to be investigated. The purpose of our research in this paper is to investigate the impact from different heating conditions and inlet steam contents on steam bubble and coolant temperature distributions in ADS fuel assemblies during a postulated SGTR accident by performing necessary computational fluid dynamics (CFD) simulations. In this research, the open source CFD calculation software OpenFOAM, together with the two-phase VOF (Volume of Fluid) model were used to simulate the steam bubble behavior in heavy liquid metal flow. The model was validated with experimental results published in the open literature. Based on our simulation results, it can be noticed that steam bubbles will accumulate at the periphery region of fuel assemblies, and the maximum temperature in fuel assembly will not overwhelm its working limit during the postulated SGTR accident when the steam content at assembly inlet is less than 15%.

## 1. Introduction

Nuclear energy, as a type of clean energy, can effectively reduce carbon dioxide emissions and therefore the greenhouse effect. At present, the fourth-generation nuclear technology that can provide cleaner and safer nuclear power has attracted gradually more attention. In the current nuclear industry, nuclear waste disposal is considered to be one of the most significant problems [1]. During the International Atomic Energy Agency Conference, six advanced nuclear reactor systems were proposed as Generation-IV reactor designs, which were considered to be able to partially solve this problem. Lead-based fast reactors [2,3,4], as one of these reactor types, have been proved to be able to efficiently transmute long-life radioactive nuclides in nuclear waste thanks to their excellent inherent safety characteristics [5,6].

At present, plenty of countries have proposed their own lead-based fast reactors (LFR) and accelerator-driven subcritical systems (ADS) designs, as listed in Table 1.

ADS was considered one of the most promising devices to perform nuclear waste transmutation, which applies an accelerator to provide a high-energy and high-current proton beam, which bombards heavy nuclei to produce high-flux broad-spectrum spallation neutrons. This external neutron source can drive and maintain the continuous and stable operation of the subcritical core. The reactor can burn nuclear waste and convert long-life nuclides to short-life nuclides with sufficient margin to core failure thanks to its inherent safety characteristics. After more than 20 years of preliminary research, at the end of 2015, the China initiative accelerator-driven system (CiADS) project was approved. The CiADS project adopts the technical route of the combination of a “superconducting linear accelerator + high-power spallation target + subcritical reactor”. Its conceptual design has been completed, and a series of key scientific and technical research is still ongoing.

In the CiADS project, a lead-bismuth eutectic (LBE) was proposed as the primary coolant thanks to its good neutron economy and suitable thermophysical properties [10]. Its primary loop mainly consists of core, primary pumps and steam generators [11]. In each steam generator unit, a large number of pipes are installed. In the primary side of pipe wall, the primary coolant LBE works at a pressure slightly higher than the atmosphere pressure, while the water in the secondary side of pipe works at a much higher pressure aiming at a better heat transfer efficiency. This may cause a steam intrusion into the primary side or even into the core region of CiADS when the steam generator tube ruptures (SGTR) accident happens [12]. Therefore, a safety analysis towards the SGTR accident should be considered when designing the CiADS [13,14].

With the continuous development of high-performance computers and numerical calculation methods, computational fluid dynamics (CFD) has been widely used in validating various ADS designs [15]. Chen [16] studied the bundle in single phase and simulated the pin bundle blockage in a fuel assembly. Koloszar [17] used the CFD model to investigate the flow pattern in the core region of MYRRHA. Zhang [13] simulated the two-phase flow in a heavy liquid metal pool and assessed bubble-risen behaviors in it. Jeltsov [14] simulated the two-phase flow in a heat-exchanger and found the SGTR accident may be worse when the broken area is close to the primary pump.

OpenFOAM is an excellent CFD analysis tool based on the finite volume method and written with C++ [18]. Many researchers are using OpenFOAM to perform complex flow simulations thanks to its editable solver and flexibility in governing equation settings.

The VOF method proposed by Hirt and Nichols [19] in 1981 has the advantages of fewer iterations and a higher accuracy when performing two-phase simulations because of the application of the Euler–Euler multiphase model and the fluid volume setting method. Zhu [20] performed a systematic study on the formation of droplets in gas microchannels using the VOF method. Chen [21] and others used the VOF method to simulate the interaction process of the water–air interface. Li [22,23,24] carried out a numerical simulation of the movement characteristics of a single bubble in a gas–liquid two-phase flow under high pressure. VOF method was proved to be suitable for simulating the multiphase flow between a variety of immiscible fluids by setting the gas-phase content in each grid.

In recent CFD studies, the k-epsilon model, the k-omega model, the SST model and the LES method were introduced to predict the turbulence flow. The k-epsilon model and the k-omega model are RANS models, which can provide higher calculation efficiency, but less accuracy in some cases [25]. The SST model was reported to be able to provide high accuracy for a low-Reynolds flow [26]. The LES model could predict the fluid more accurately than RANS, but with a higher requirement for calculation resources [25]. The MYRRHA project [17] and Sugrue [18] recommended to use the k-epsilon model for two-phase LBE flow simulations. Therefore, we considered it to be suitable for the studies performed in this paper [27,28,29,30]. Waite [31] reported that the simulation results calculated with the k-epsilon model for the two-phase flow in rod bundles were accordant with experimental results. The standard k-epsilon model was then chosen for the turbulence simulations performed in this study.

In recent studies, the first-order upwind scheme, the second-order upwind scheme, the central difference scheme and the QUICK scheme were performed. The first-order upwind scheme is widely used in engineering for its efficiency, but it may be not accurate. The central difference scheme and the QUICK scheme may be unstable. The second-order upwind scheme has the intercept of second-order accuracy, but it still has the problem of false diffusion. According to the studies performed by other researchers [18,32], in this work, the second-order upwind convection scheme was adopted. Time integration was performed using the first-order method. The PIMPLE algorithm was used as the pressure-velocity coupling scheme, which is a combination of the PISO and the SIMPLE algorithm by OpenFOAM.

In this paper, the OpenFOAM based on the VOF method was adopted to study the two-phase flow under different inlet and heating conditions. In Section 2, the simulation model was created, including implementing the governing equations of VOF, meshing with ADS configurations and setting the coolant properties and boundary conditions. The validation of our simulation model was also included in this section. In Section 3, the simulation results of different inlet steam contents and heating conditions are summarized and discussed.

## 2. Materials and Methods

### 2.1. Governing Equations

In the VOF model, gas phase is considered to have the same velocity as the liquid phase. Among them, the mixed density and mixed dynamic viscosity are calculated as:

(1) $$\rho =\alpha \rho_{l}+(1-\alpha )\rho_{g}$$

(2) $$\mu =\alpha \mu_{l}+(1-\alpha )\mu_{g}$$

The governing equation of α is:

(3) $$\frac{\partial \alpha }{\partial t}+\nabla \cdot (\alpha U)=0$$

The mass equation can be described as:

(4) $$\frac{\partial \rho }{\partial t}+\nabla \cdot (\rho U)=0$$

The momentum equation can be described as:

(5) $$\frac{\partial \rho U}{\partial t}+\nabla \cdot (\rho UU)-\nabla \cdot \tau =-\nabla p+\rho g+\sigma \kappa \nabla \alpha$$

The energy equation can be described as:

(6) $$\frac{\partial \rho T}{\partial t}+\nabla \cdot (\rho UT)-\nabla \cdot \left(\frac{k_{f}}{c_{p}}\nabla T\right)=0$$

The equation of turbulence kinetic energy *k* is:

(7) $$\frac{\partial \left(\rho k\right)}{\partial t}+\nabla \cdot (\rho Uk)=\nabla \cdot \left[\left(\mu_{m}+\frac{\mu_{t}}{\sigma_{k}}\right)\nabla k\right]+G_{k}-\rho \varepsilon$$

The equation of turbulence kinetic energy dissipation epsilon is

(8) $$\frac{\partial \left(\rho \varepsilon \right)}{\partial t}+\nabla \cdot (\rho U\varepsilon )=\nabla \cdot \left[\left(\mu_{m}+\frac{\mu_{t}}{\sigma_{\varepsilon }}\right)\nabla \varepsilon \right]+\frac{\varepsilon }{k}\left(C_{\varepsilon 1}G_{k}-C_{\varepsilon 2}\rho \varepsilon \right)$$

Then, Equations (7) and (8) are coupled by the following equations:

(9) $$\mu_{t}=\rho C_{\mu }k^{2}/\varepsilon$$

(10) $$G_{k}=\mu_{t}\nabla U\cdot \left[\nabla U+{\left(\nabla U\right)}^{T}\right]$$

where $\mu_{m}$ is the dynamic viscosity of laminar fluid; $\mu_{t}$ is the dynamic viscosity of turbulent fluid; $\sigma_{k}$ and $\sigma_{\varepsilon }$ represent diffusion Prandtl numbers ($\sigma_{k}=1.0$ and $\sigma_{\varepsilon }=1.3$); the model constants $C_{\mu }$, $C_{\varepsilon 1}$ and $C_{\varepsilon 2}$ are 0.09, 1.44 and 1.92, respectively.

### 2.2. Meshing the ADS Assembly

**Figure 1:**
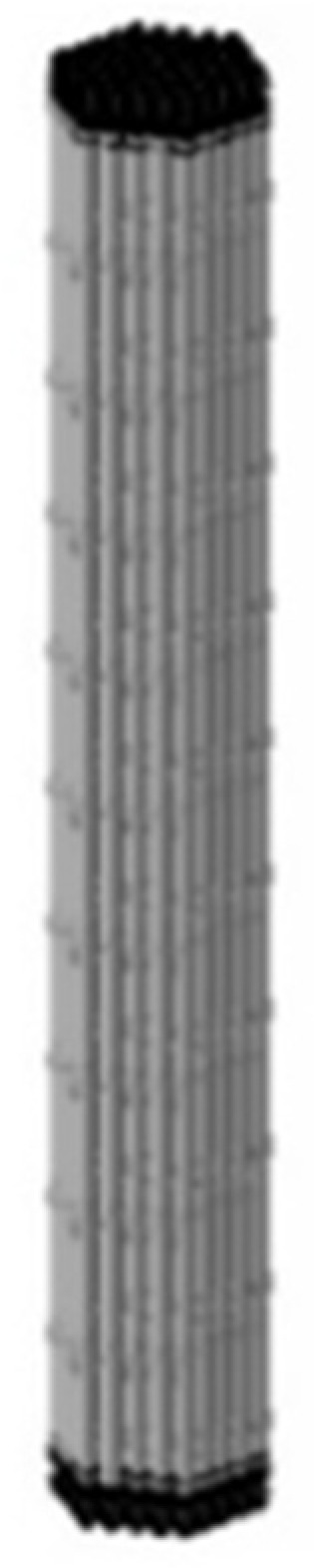
The 3D structure of the fuel assembly.

The ADS project adopts a subcritical reactor, whose thermal power is 8 MW. Fuel assemblies in the ADS subcritical core adopt a regular hexagonal outer tube encapsulation structure. The pin diameter is 6.55 mm and the fuel rod center distance is 9.17 mm. In order to prevent the fuel assembly from floating in the lead–bismuth coolant, the counterweight and the locking mechanism are installed at the lower end of the fuel assembly. The 3D structure of the fuel assembly is shown in Figure 1.

**Figure 2:**
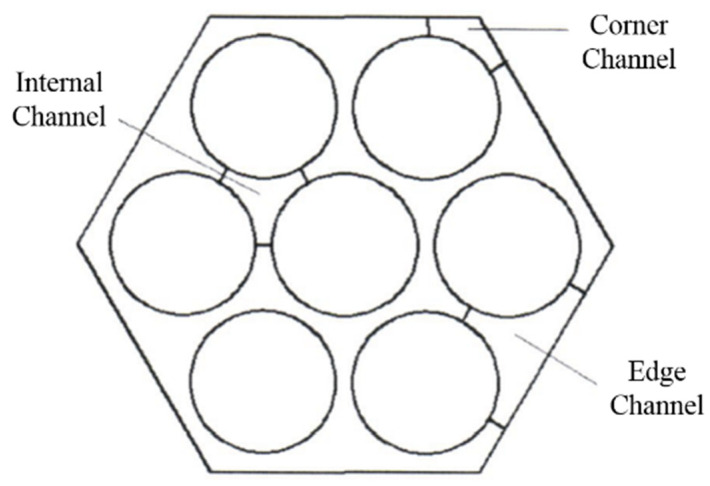
Fuel assembly subchannels.

As shown in Figure 2, the flow channel in the fuel assemblies can be subdivided as internal channels, edge channels and corner channels based on their different heat transfer characteristics. The internal channel is heated by three adjacent one-sixth fuel rods, whose total heating area is a half fuel rod. The edge channel is heated by two-quarter fuel rods, whose total heating area is a half fuel rod, and one patch is an adiabatic plane. The corner channel is heated by a one-sixth fuel rod, and two patches are adiabatic planes. These differences in heating area and hydraulic diameters will affect the coolant flow patterns in the channels.

**Figure 3:**
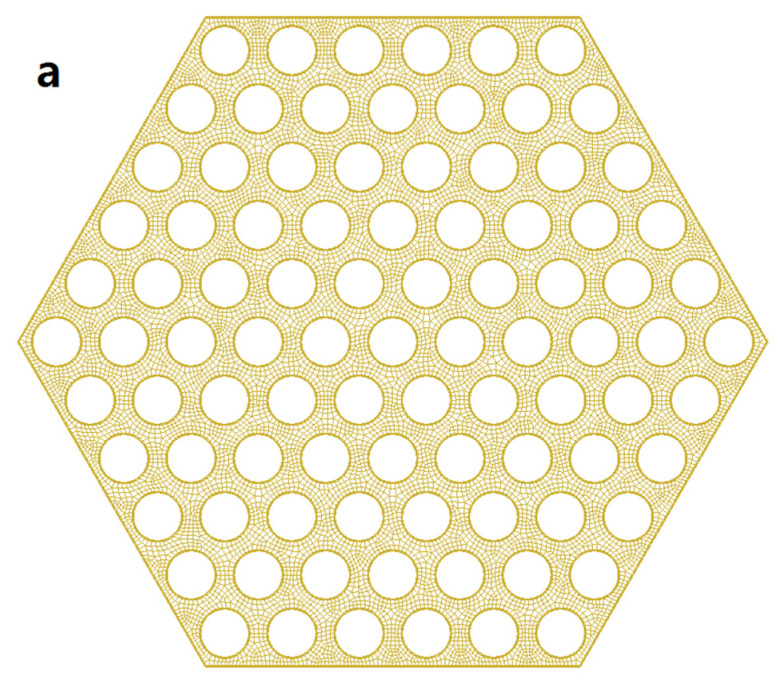

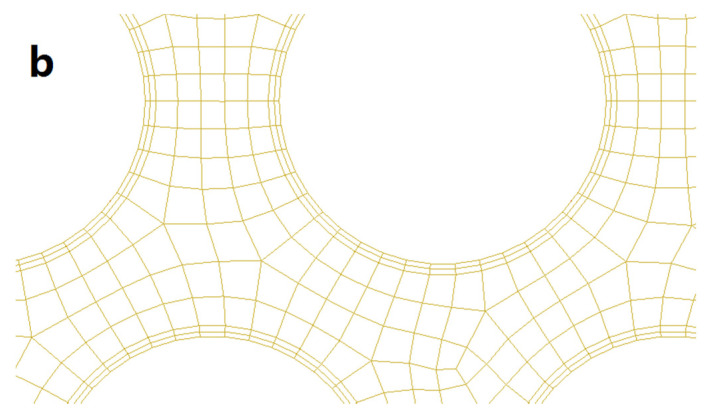
Grid division of an assembly (**a**) and boundary zones (**b**).

**Figure 4:**
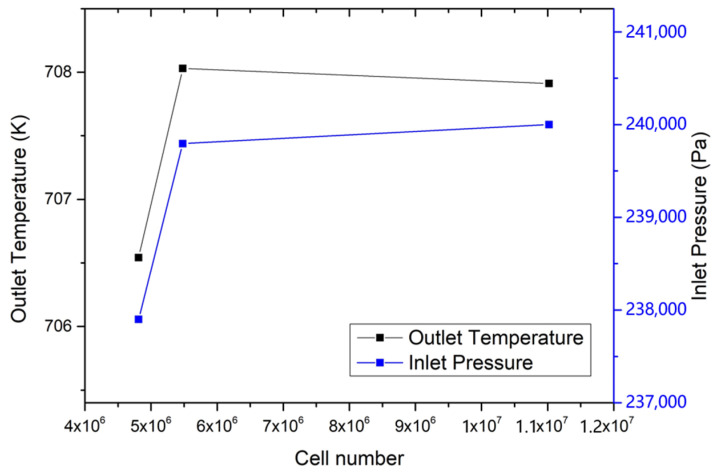
Grid convergence study.

Figure 3 shows the grid division on the Z plane. The total number of cells is 5,478,900, the max skewness is 0.79, the mesh orthogonal quality is higher than 0.7 and the y-plus value is 30. To reduce the computational complexity, the space wire was neglected from the grid division and the grid therefore was a hexahedral grid. A coarse mesh with 4,812,000 cells and a fine mesh with 11,022,000 cells were built to compare with the 5,478,900 cells in this study. After given the same boundary condition, the simulation results with the 5,478,900 cells were reported to be close to those with the 11,022,000 cells as shown in Figure 4. The simulation was conducted using a Dell Server 7920, which has 80-core CPUs and 128GB RAM. For a simulation of a 10 s scenario, the computing time of meshes with 4,812,000 cells, 5,478,900 cells and 11,022,000 cells were 30.5 h, 42.3 h and 103.2 h, respectively. Therefore, considering the computational economy and accuracy, the mesh with 5,478,900 cells was chosen for this study.

### 2.3. Coolant Properties

The thermophysical parameters of lead–bismuth eutectic (LBE) and lead (Pb) coolant have been experimentally measured and fitted into correlations as listed in Table 2 [33].

The steam properties obtained from the international standard IAPWS-IF97 steam table were defined into the OpenFOAM material database [34]. These steam properties were reported to be valid in the temperature region of 273.15 K–2273.15 K and for pressure lower than 10 MPa.

### 2.4. Boundary Conditions Setting

**Figure 5:**
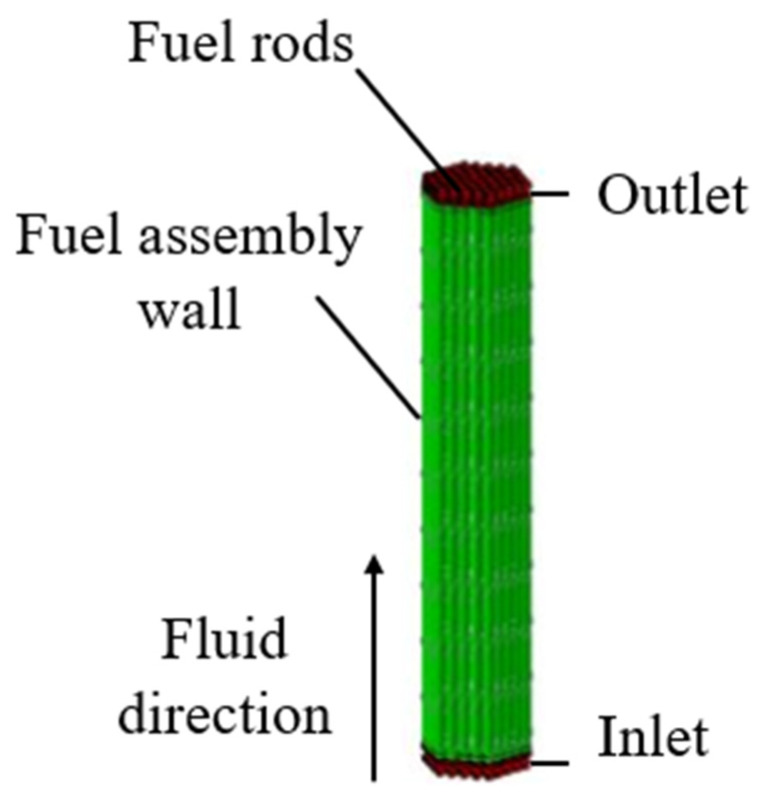
Boundary condition settings.

Figure 5 shows the boundary condition settings of the fuel assembly. The inlet velocity of fluid was defined as 0.2 m/s with the direction vertical to the patch. The wall was defined as a no-slip wall [35]. The outlet flow condition was defined as a pressure outlet equal to the atmosphere pressure. The transient mode was adopted to simulate bubble behaviors in the fuel assembly.

To simulate the distribution of bubbles in the fuel assembly under different boundary conditions, simulations were divided into 4 cases as listed in Table 3.

### 2.5. Verification of Our Simulation Model

**Figure 6:**
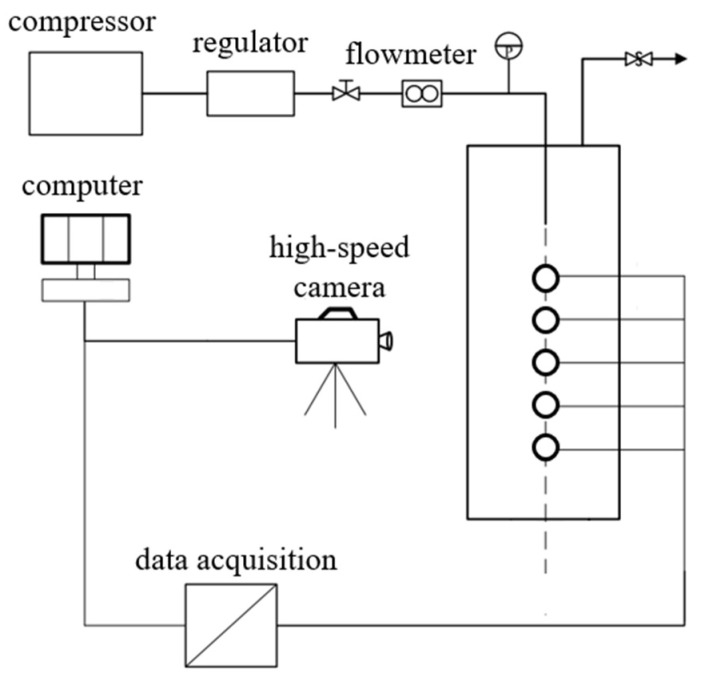
Schematic diagram of the experimental apparatus.

The migration of steam bubbles in heavy liquid metal is complicated. In this process, bubbles are subjected to buoyancy, surface tension, drag force and other effects. The floating process cannot be optically observed, nor can it be observed by X-ray penetration due to the high density and opacity of the heavy liquid metal. To verify our simulation model, we referred to the experimental results obtained with an experimental device [13], in which high-speed gas was injected into the water tank to form a two-phase flow and recorded by the high-speed camera. Figure 6 shows the set-up of this experimental device, which includes a gas injection system, a water tank, a high-speed camera, sensors and measurement systems. The high-pressure air produced by the compressor was injected into the water tank through a pipe. The diameter of the pipe nozzle is 4 mm and it was located at 5 mm under the water. The water tank is a glass cuboid with a length of 250 mm, a width of 250 mm, and a height of 800 mm. The high-speed camera is used to observe the bubble shape and the depth of injection. During the experiment, the gas injection velocity was set as 22.12 m/s, 26.54 m/s, 33.18 m/s, 35.39 m/s, 44.24 m/s, 55.30 m/s, 66.36 m/s, 77.42 m/s, 88.48 m/s and 99.54 m/s, respectively.

In order to model this experiment into OpenFOAM, we first constructed a model of the water tank. The surrounding walls and the bottom of the tank were defined as no-slip walls in our simulation model. The top of the tank was defined as an outflow patch at the ambient pressure. The nozzle was modeled in the center of the top patch. The computational area was divided into 250, 250 and 800 meshes in the X, Y and Z directions. The model was constructed based on the geometrical details of the experiment tank.

**Figure 7:**
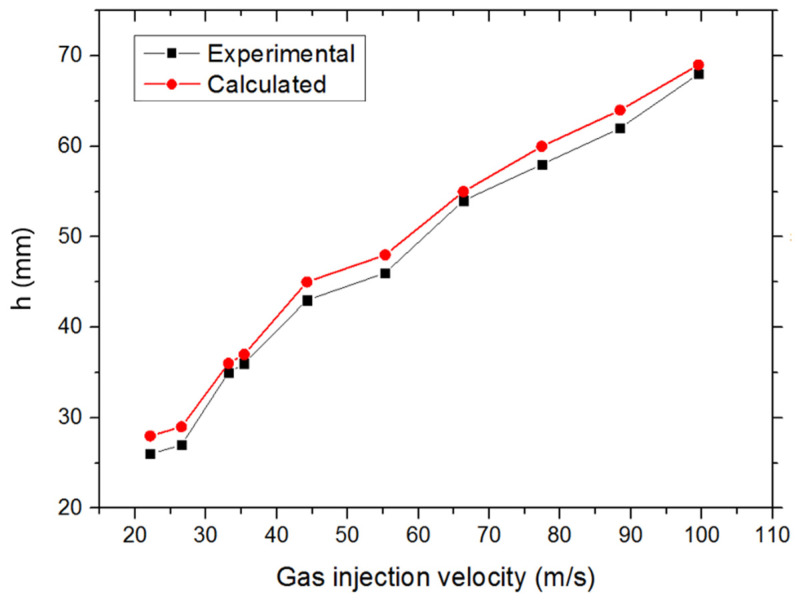
Bubble penetration depth for the gas injection into water.

Figure 7 shows the results from the experiments and the simulations. As can be seen from Figure 7, the experimental and simulation results are in good agreement. The maximum relative error (approximately 7.7%) occurred at the velocity of 22.12 m/s, which is considered to be mainly caused by the small denominator. The absolute differences between the experimental and the simulated results were reported to be less than 2 mm. Based on the results stated above, the VOF model was considered to be suitable for simulations performed in the following sections.

## 3. Simulations under Different Boundary Conditions

### 3.1. Temperature Distributions at the Middle Section and the Outlet Section

**Figure 8:**
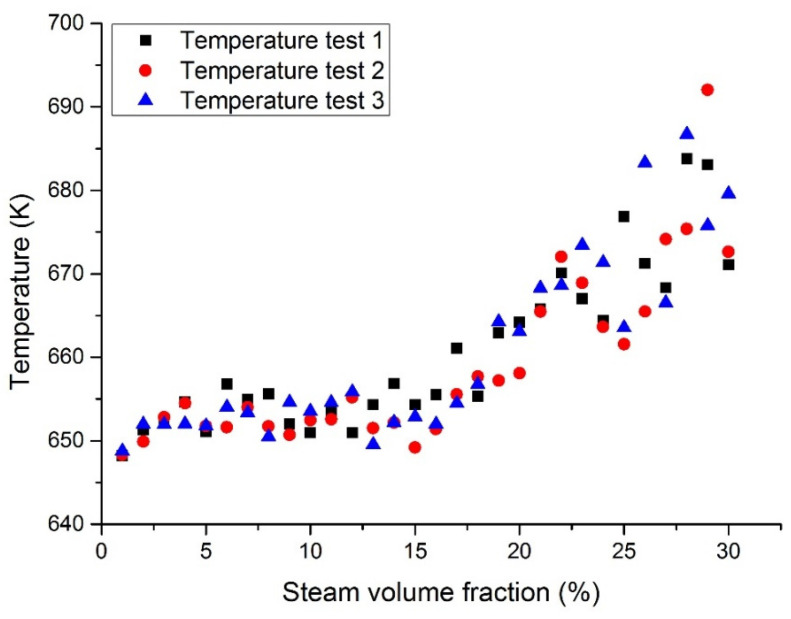
Maximum LBE temperature at the assembly middle section with a fixed heat flux (case 1).

**Figure 9:**
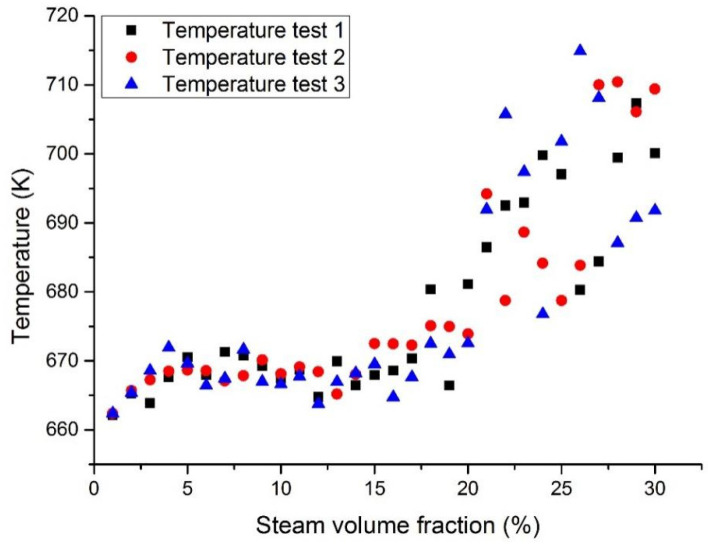
Maximum Pb temperature at the assembly middle section with a fixed heat flux (case 3).

Figure 8 and Figure 9 show the maximum temperatures at the middle section in case 1 and case 3. The middle section was set in parallel with the inlet section and allocated at the center of the fuel assembly. When the coolant was pure LBE coolant, the maximum temperature at the middle section was reported to be 646 K. When the coolant was pure liquid Pb, the maximum temperature at the middle section was 651 K. This means that the designed temperature of the Pb coolant reactor was slightly higher than that of the LBE coolant. When the inlet steam content increases from 1 to 15%, the temperature at the middle section will slowly increase. When the inlet steam content was less than 15%, the temperature in the reactor fuel assembly was reported to be lower than its working limit. When the inlet steam content exceeds 15%, the temperature will rise significantly and the temperature fluctuations will also become larger.

**Figure 10:**
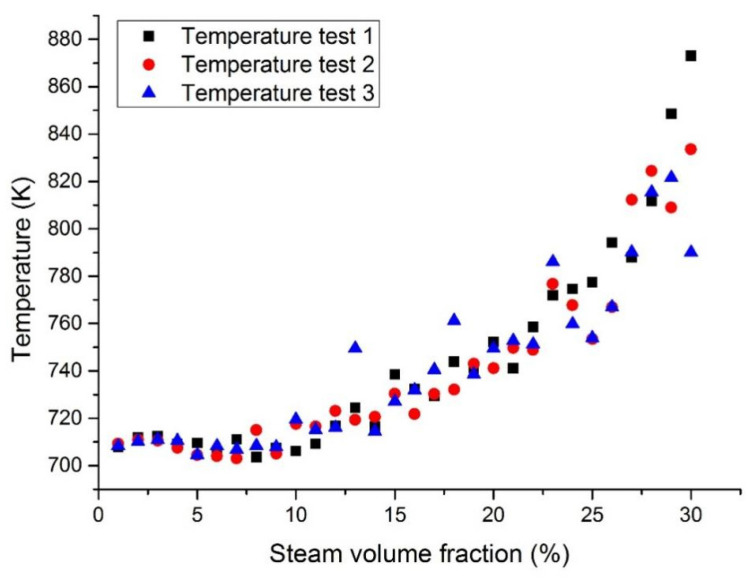
Maximum LBE temperature at the assembly outlet section with a fixed heat flux (case 1).

**Figure 11:**
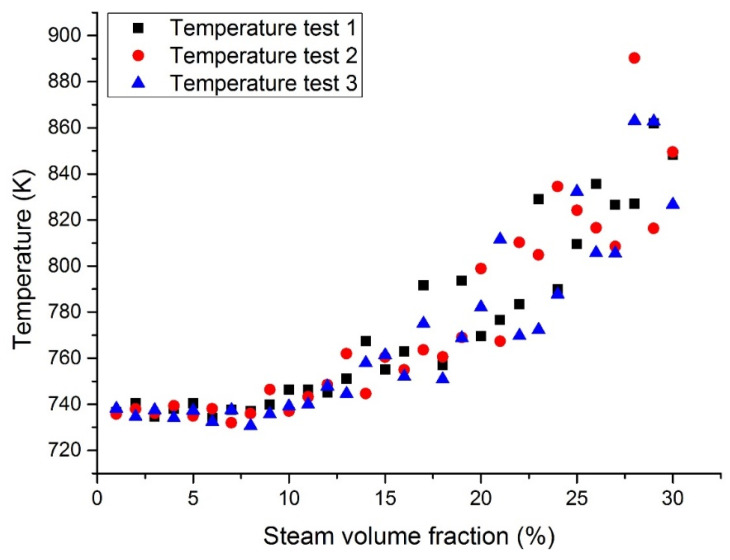
Maximum Pb temperature at the assembly outlet section with a fixed heat flux (case 3).

Figure 10 and Figure 11 show the maximum temperatures at the outlet section in case 1 and case 3. According to the three test results, the maximum temperature of liquid Pb was higher than that of the lead–bismuth coolant with the same inlet steam content. When a large number of bubbles enter, the temperature fluctuation of the Pb coolant is greater than that of the lead–bismuth coolant. When the inlet steam content is less than 15%, the calculated fluid temperature is lower than its working limit. It was reported that the fuel bundle may burn up when the inlet steam content exceeds 15% due to the high cladding temperature incurred by insufficient cooling.

### 3.2. Bubble Gathering

**Figure 12:**
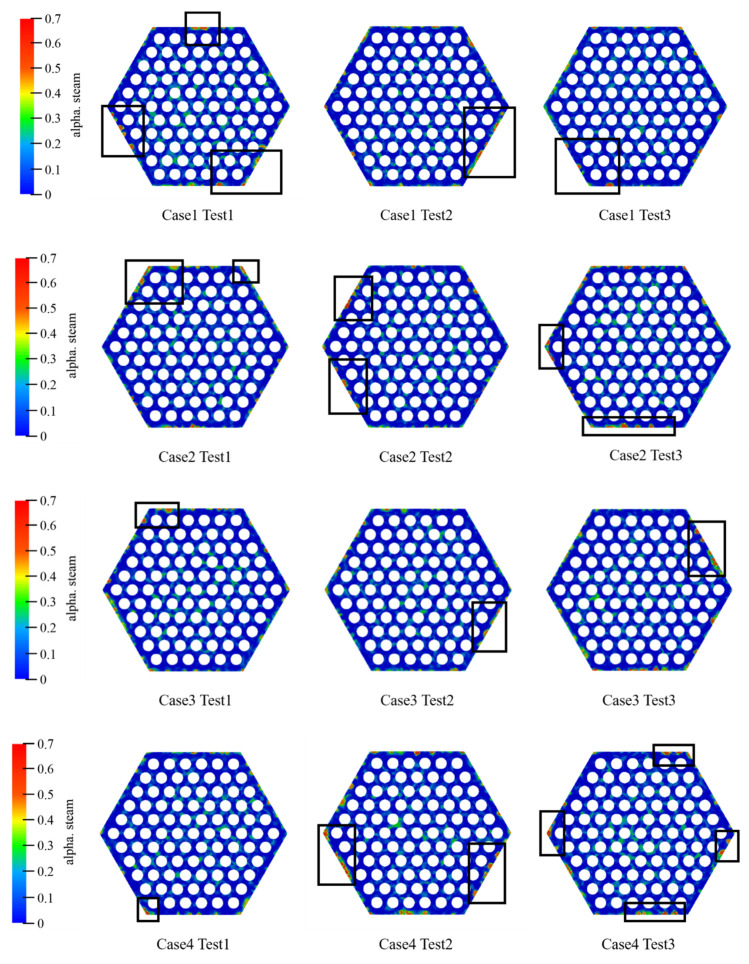
Bubble distribution in the cases 1 to 4 with 10% inlet steam content. The α steam stands for the steam content.

**Figure 13:**
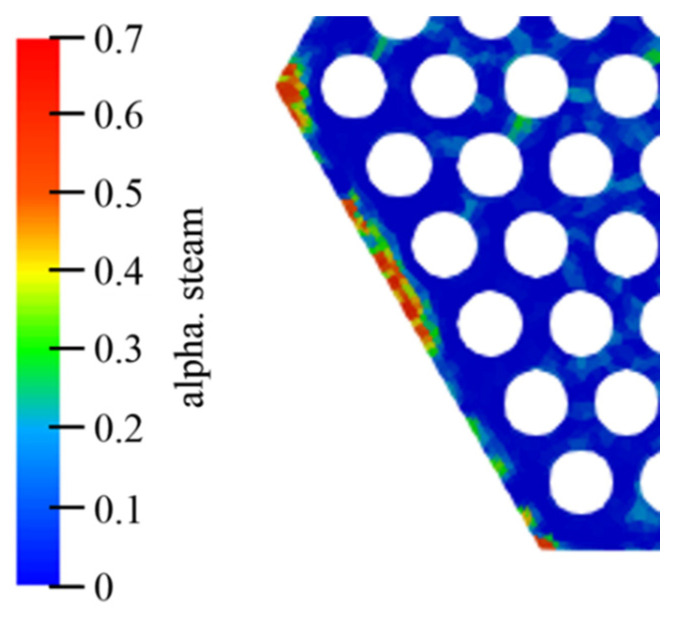
Steam bubbles at the periphery regions.

Figure 12 shows the distribution of the gas phase when the inlet steam content is 10% in each case. Figure 13 shows the bubble accumulation at the assembly outlet section with a fixed tube wall temperature. It can be noticed that the steam bubbles will accumulate at the periphery regions. The probability of the largest bubble appearing in the corner channel exceeds 50%. This phenomenon may cause fuel rods to operate under risky conditions. The bubble accumulation may be caused by the larger fluid velocity in the internal channel than in the periphery channel.

### 3.3. Outlet Section Velocity

**Figure 14:**
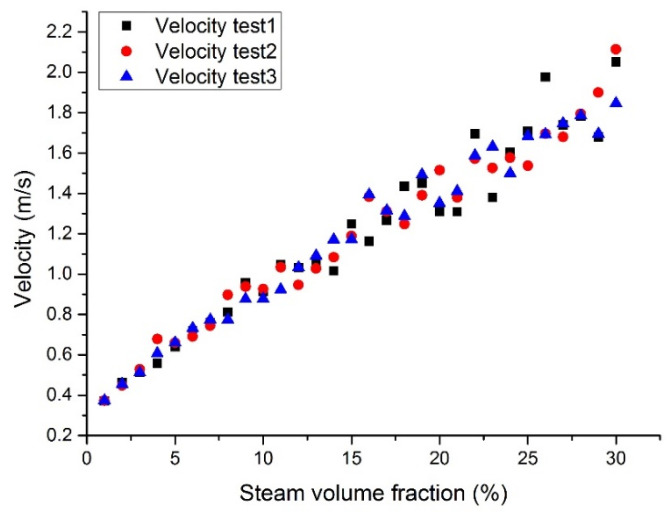
Maximum LBE velocity at the assembly outlet section with a fixed heat flux (case 1).

**Figure 15:**
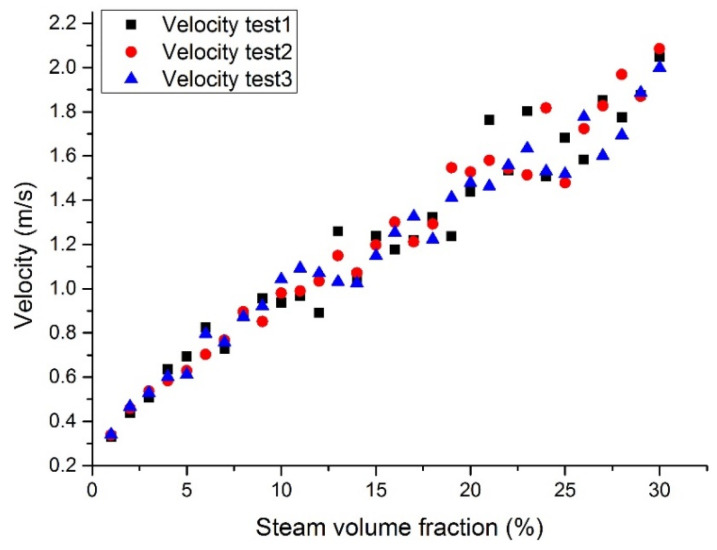
Maximum LBE velocity at the assembly outlet section with a fixed tube wall temperature (case 2).

**Figure 16:**
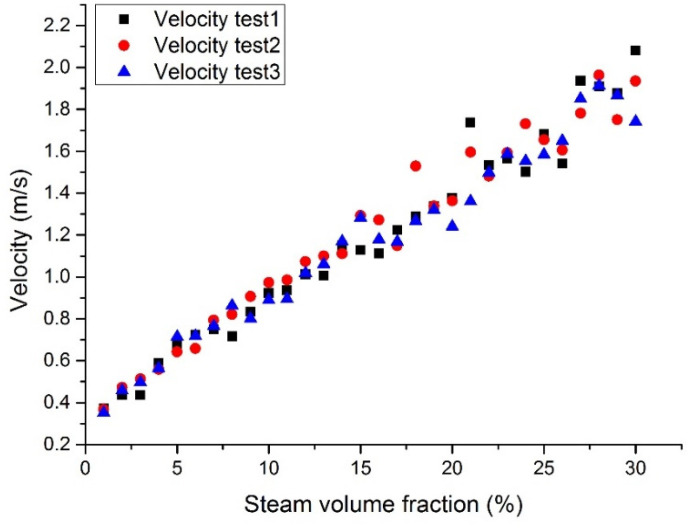
Maximum Pb velocity at the assembly outlet section with a fixed heat flux (case 3).

**Figure 17:**
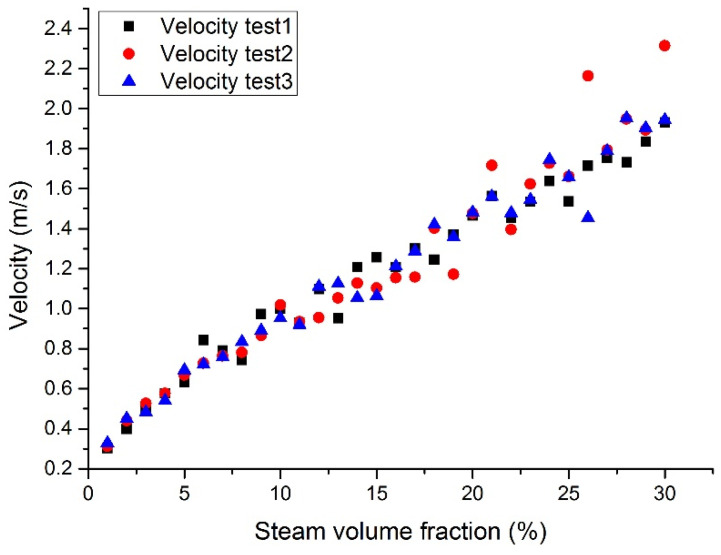
Maximum Pb velocity at the assembly outlet section with a fixed tube wall temperature (case 4).

Figure 14, Figure 15, Figure 16 and Figure 17 show the maximum velocity at the outlet section. It can be seen from these figures that the maximum velocity gradually increases with the increase of the inlet steam content. Together with the outlet section temperatures, it can be noticed that the temperature has little effect on velocity, mainly because the steam content plays a leading role in velocity. Due to the steam expansion, the two-phase flow velocity will rise with the inlet steam content.

### 3.4. Maximum Steam Volumetric Fraction

**Figure 18:**
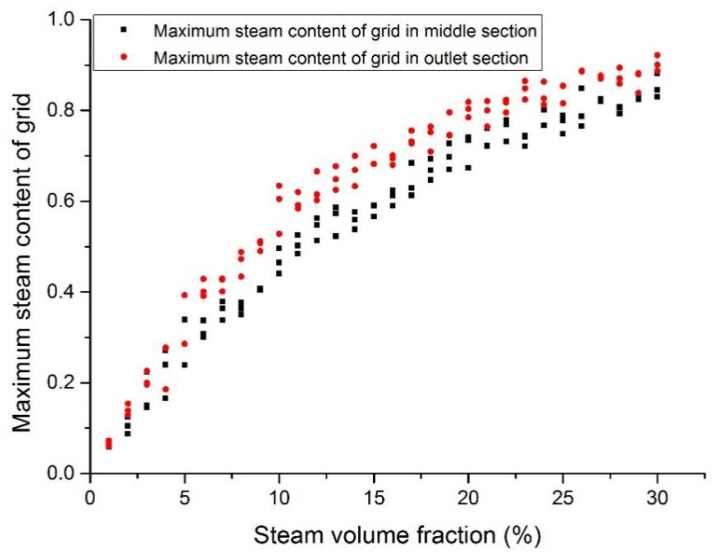
Maximum steam content at the assembly outlet section (case 1).

**Figure 19:**
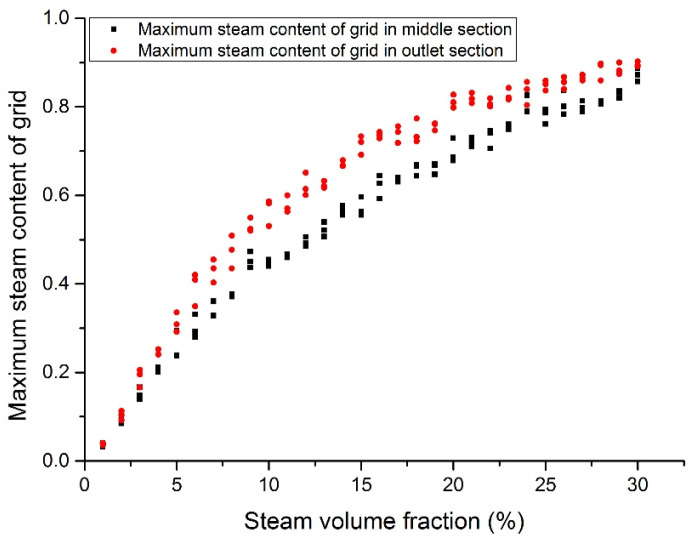
Maximum steam content at the assembly outlet section (case 2).

**Figure 20:**
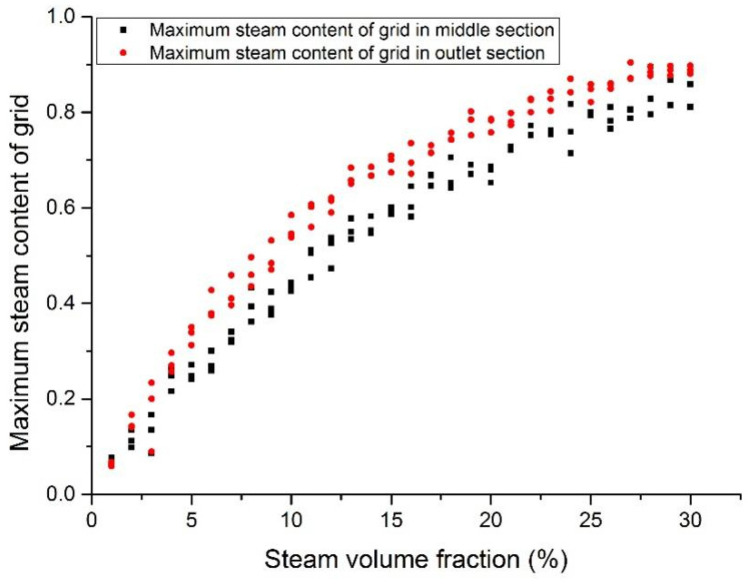
Maximum steam content at the assembly outlet section (case 3).

**Figure 21:**
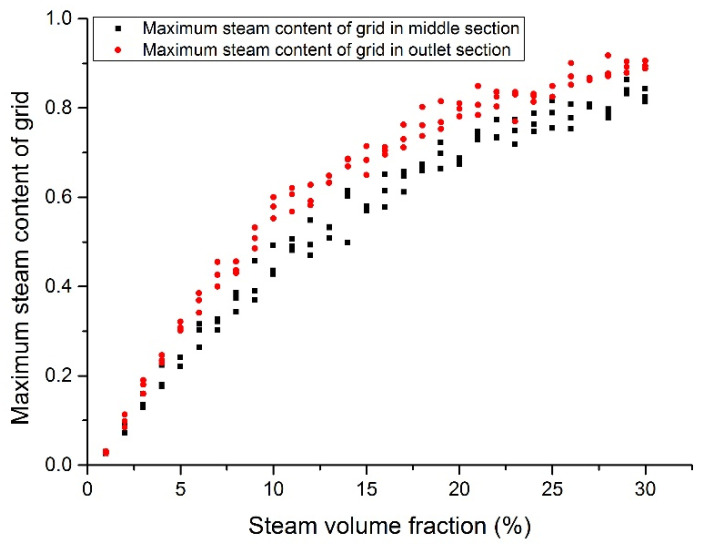
Maximum steam content at the assembly outlet section (case 4).

Figure 18, Figure 19, Figure 20 and Figure 21 show the maximum grid steam volumetric content at the middle section and the outlet section. The steam content at the middle section is lower than that at the outlet section. The steam content in the grid increases significantly from 5 to 15%. When the inlet steam content is higher than 15%, the floating speed slows down because of the occupation of steam bubbles in the upper region. When the inlet steam content is less than 2%, the grid steam content at the middle section and the outlet section are basically the same. When the inlet steam content increases from 5 to 20%, the grid steam content at the middle section and the outlet section are significantly different. It was reported that the bubbles would break and merge together under this condition, which may cause fluctuations of fuel rods temperature and therefore thermal fatigue. When the inlet steam content exceeds 20%, the difference becomes smaller.

## 4. Conclusions

In this paper, the open source CFD software OpenFOAM was adopted to simulate the two-phase heavy liquid metal flow. Based on our simulation results, the following points can be preliminarily concluded:

(1) When the steam content is lower than 15%, the coolant temperature rises slowly. The fuel rod would operate within its designed limit. A significant rise of coolant temperature may be noticed after the inlet steam content exceeds 15%.
(2) The temperature fluctuation in liquid Pb (lead) due to the steam bubble intrusion was reported to be slightly higher than that in LBE coolant.
(3) The temperature fluctuation is significant when the inlet steam content is higher than 15%, which may cause the failure of fuel assemblies. It was reported that this significant phenomenon needs to be carefully investigated.
(4) Steam bubbles tend to accumulate at the periphery regions, especially in the corner channels. This phenomenon confirms the necessity of constructing corresponding experimental facilities in the future.
(5) The outlet fluid velocity and the steam content were reported to be mainly affected by the inlet steam content.

## Figures and Tables

**Table 1:** Coolants in typical lead-based fast reactors (LFRs) designs [6,7,8,9].

Reactor	Coolant	Country
SVBR-100	LBE	Russia
SSTAR	Pb	American
LSFR	LBE	Japan
CiADS	LBE	China
CLEAR-SR	LBE	China
MYRRHA	LBE	Belgium
SEALER	Pb	Sweden

**Table 2:** Properties of liquid lead–bismuth eutectic (LBE) and Pb.

Properties	Liquid LBE	Liquid Pb
$\rho \left[kg/m^{3}\right]$	11096−1.3236T	11367−1.1944T
μ[Pa⋅s]	$4.94\cdot 10^{-4}\cdot \exp\left(754.1/T\right)$	$4.55\cdot 10^{-4}\cdot \exp\left(1069/T\right)$
$c_{p}\left[\frac{J}{kg\cdot K}\right]$	$159.0-0.0272T+7.12\cdot 10^{-6}{\left(T\right)}^{2}$	$161.5-0.0268T+6.98\cdot 10^{-6}{\left(T\right)}^{2}$
$\lambda \left[\frac{W}{m\cdot K}\right]$	$3.61+1.517\cdot 10^{-2}T-1.741\cdot 10^{-6}{\left(T\right)}^{2}$	9.2+0.011T

**Table 3:** The setting of different cases.

Case Number	Coolant	Heating Boundary Condition
Case 1	LBE	Fixed Heat Flux value
Case 2	LBE	Fixed Wall Temperature
Case 3	Pb	Fixed Heat Flux value
Case 4	Pb	Fixed Wall Temperature

## Data Availability

Data sharing not applicable.

## Nomenclature

**Symbol**	**Explanation**
ρ, $\rho_{l}$ and $\rho_{g}$	Mixed Fluid Density, Liquid Density and Gas Density (kg·m^−3^)
α	volume fraction of fluid
μ, $\mu_{l}$ and $\mu_{g}$	mixed fluid dynamic viscosity, liquid dynamic viscosity and gas dynamic viscosity (Pa·s)
t	time (s)
U	Gas phase and liquid phase mixed velocity (m·s^−1^)
τ	viscous stress (Pa)
p	pressure (Pa)
g	gravity (m·s^−2^)
σ	surface tension coefficient (N·m^−1^)
κ	curvature (m^−1^)
T	temperature (K)
k_f_	fluid heat transfer coefficient (W·m^−2^·K^−1^)
$c_{p}$	specific heat capacity at constant pressure (J·kg^−1^·K^−1^)
λ	thermal conductivity (W·m^−1^·K^−1^)
